# Intracellular hyaluronic acid-binding protein 4 (HABP4): a candidate tumor suppressor in colorectal cancer

**DOI:** 10.18632/oncotarget.27804

**Published:** 2020-11-17

**Authors:** Talita Diniz Melo-Hanchuk, Carolina Colleti, Ângela Saito, Maria Carolina Santos Mendes, José Barreto Campello Carvalheira, Jose Vassallo, Jörg Kobarg

**Affiliations:** ^1^School of Pharmaceutical Sciences, State University of Campinas (UNICAMP), Campinas, SP, Brazil; ^2^Department of Biochemistry and Tissue Biology, Institute of Biology, State University of Campinas (UNICAMP), Campinas, SP, Brazil; ^3^Brazilian Biosciences National Laboratory (LNBio), Brazilian Center for Research in Energy and Materials (CNPEM), Campinas, SP, Brazil; ^4^Division of Oncology, Department of Internal Medicine, Faculty of Medical Sciences, State University of Campinas (UNICAMP), Campinas, SP, Brazil; ^5^Laboratory of Investigative Pathology, CIPED, Faculty of Medical Sciences, State University of Campinas, Campinas, SP, Brazil; ^*^These authors contributed equally to this work

**Keywords:** colon cancer, regulatory protein, gene knockout, proliferation, tumor marker

## Abstract

Hyaluronic Acid-binding protein 4 (HABP4) is a regulatory protein of 57 kDa that is functionally involved in transcription regulation and RNA metabolism and shows several characteristics common to oncoproteins or tumor suppressors, including altered expression in cancer tissues, nucleus/cytoplasm shuttling, intrinsic lack of protein structure, complex interactomes and post translational modifications. Its gene has been found in a region on chromosome 9q22.3-31, which contains SNP haplotypes occurring in individuals with a high risk for familial colon cancer. To test a possible role of HABP4 in tumorigenesis we generated knockout mice by the CRISPR/Cas9 method and treated the animals with azoxymethane (AOM)/dextran sodium sulfate (DSS) for induction of colon tumors. *HABP4*–/– *mice*, compared to wild type mice, had more and larger tumors, and expressed more of the proliferation marker proteins Cyclin-D1, CDK4 and PCNA. Furthermore, the cells of the bottom of the colon crypts in the *HABP4*–/– *mice* divided more rapidly. Next, we generated also HABP4*–*/– HCT 116 cells, in cell culture and found again an increased proliferation in clonogenic assays in comparison to wild-type cells. Our study of the protein expression levels of HABP4 in human colon cancer samples, through immunohistochemistry assays, showed, that 30% of the tumors analyzed had low expression of HABP4. Our data suggest that HABP4 is involved in proliferation regulation of colon cells *in vitro* and *in vivo* and that it is a promising new candidate for a tumor suppressor protein that can be explored both in the diagnosis and possibly therapy of colon cancer.

## INTRODUCTION

Colon cancer is an important contributor to worldwide cancer morbidity and mortality. It is the third most common cancer diagnosed in the United States, with an estimated 104,610 new cases and 43,340 deaths for 2020 [1]. The development of colon cancers has been related to both somatic and germline mutations [2, 3]. In an analysis of familial colon cancer risk-associated SNP haplotypes, the human gene encoding the protein intracellular hyaluronic acid-binding protein 4 (HABP4) [4], has been found in linkage disequilibrium [5].

The HABP4 was identified by cross-reactivity with Ki-1 (which recognizes CD30, =TNFRSF8), the first monoclonal antibody to identify neoplastic Hodgkin and Stenberg-Reed cells in Hodgkin’s lymphoma [6, 7]. The Ki-1 antibody recognizes aside the surface CD30 transmembrane protein a 57 kDa cytoplasmic and nuclear antigen [8, 9]. The full length cDNA cloning revealed next, that this 57 kDa protein, rich in positively charged amino acids in its sequence, interacts *in vitro* with negatively charged macromolecules such as RNAs and hyaluronic acid, among others, resulting in it´s denomination as Hyaluronic acid binding protein 4 (=HABP4) [4]. Several functional studies performed later, however, pointed to HABP4 as intracellular regulatory protein, that is rather involved in expression regulation on various levels [10–15].

This protein is expressed in different types of tissues and, as it´s paralog protein Serpin mRNA Binding Protein 1 (=SERBP1), involved in important cellular events, which are generally disturbed during the tumorigenesis processes. These include: regulation of genes transcription and expression [11, 12], translation [16], RNA splicing or mRNA metabolism [13, 14, 17], mitotic cell cycle, apoptosis or associated regulatory pathways [18–21]. The over-expression of SERBP1 was reported in glioblastoma, ovarian, breast, prostate, lung and colon cancer [22–26].

Despite the role of HABP4 in the tumorigenic process remains unclear, some pieces of evidence suggest that may have some role in some processes of oncogenesis. First it is an intrinsically unstructured protein [27], which interacts with p53 and with other p53 interacting proteins [19], and p53 is mutated in more than 50% of human cancers. HABP4 also interacted with the tumor suppressor PKC [20]. The over-expression of HABP4 affected the proliferation and apoptosis *in vitro* [11, 20, 28].

One of the key features of cancer is uncontrolled proliferation [29]. In human embryonic kidney HEK 293 cells, the over-expression of HABP4 not only resulted in alterations of the expression levels of genes related to proliferation and cell cycle but also led cells to a reduction of cell growth and proliferation [11].

In this study, we addressed the impact of HABP4 gene deletion, on the development of colon cancer. A *Habp4–/–* knockout mice and colon cancer cell lines were generated using the CRISPR/Cas9 gene-editing system. We hypothesized that HABP4 could modify inflammatory carcinogenesis, but its effects were unclear. To test this hypothesis, we applied the AOM/DSS treatment to *HABP4–/–* mice and analyzed the HABP4 protein expression in human tissue. We found that the absence of HABP4 leads to an increase in cell proliferation in colon epithelium and *in vitro*. We established the relevance of HABP4 for colon cancer development by demonstrating the increased number of tumors in a mouse model for colitis-associated colon cancer in *Habp4–/–*, which is convergent with the down regulation of HABP4 expression in patients with colorectal cancer.

## RESULTS

### Generation of *HABP4* gene knockout mice

**Figure 1:**
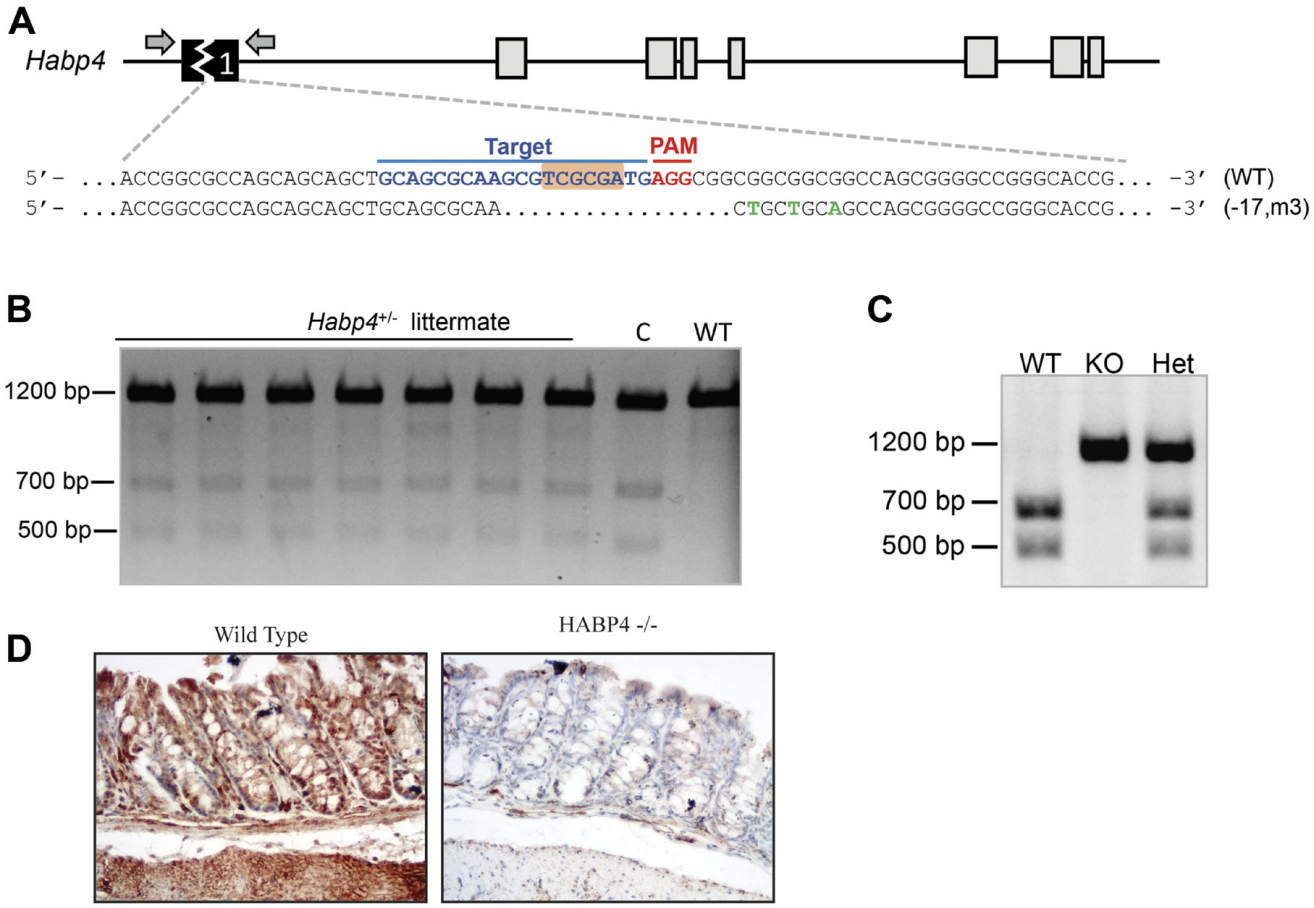
Generation of HABP4 knockout mice. (**A**) Strategy for targeting mouse *Habp4* using CRISPR/Cas9 genome editing tool. In the wild-type (WT) allele, the sgRNA target sequence is shown in blue, Protospacer Adjacent Motif (PAM) in red and the endogenous NruI restriction enzyme site is highlighted in orange. In mutant allele, a 17-bp deletion and 3-bp substitution are shown in green; (**B**) Genotyping by T7 Endonuclease I assay of heterozygous littermates. c indicates a positive heterozygous animal control and WT: is a negative control (non-altered wild-type HABP4 locus); (**C**) Different genotyping method by NruI restriction enzyme assay, discriminating the wild-type (WT), heterozygous (het) or homozygous (KO) genotypes; (**D**) Immunohistological staining confirmed the knockout of HABP4 in the colon epithelium.

To knockout mouse HABP4 gene through the CRISPR/Cas9 technique, a 20-nt sgRNA targeting the first exon of *Habp4* was designed, cloned into the px330 vector, which was subsequently injected into C57BL/6J mouse zygotes. After sequencing the target *locus* of generated animals, a 17-bp deletion and 3-bp substitution were found in the predicted region of Cas9 cleavage (Figure 1A). This mutation removed the endogenous NruI restriction enzyme site (TCGCGA) located at the sgRNA target sequence, allowing to use NruI digestion as a second genotyping method.

To decrease the influence of variations other than the gene knockout, a littermate strain was used. *HABP4* knockout mice were mated with wild-type ones for the generation of a heterozygous littermate strain (Figure 1B). These animals were intercrossed to originate wild-type, heterozygous, and knockout (*Habp4 –/–*) animals (Figure 1C). Mice were genotyped either by the T7 endonuclease I assay (Figure 1B), in which cleaved bands (500 bp and 700 bp) indicate the presence of mutant DNA, or by NruI restriction enzyme cleavage (Figure 1C), in which, to the contrary of the former test, a single 1200-bp band indicates the knockout allele, whereas the presence of 700-bp and 500-bp bands indicates the wild-type allele (Figure 1C).

Besides the genetic confirmation, knockout mice were also confirmed at the protein level. To assess this information, the colon of wild-type and knockout animals were collected and paraffinized for immunohistochemistry assays. Using an anti-HABP4 antibody, we observed expression of HABP4 protein in the wild-type mice, presenting high marking level throughout the crypt, while the knockout mice did not show staining (Figure 1D).

### HABP4 knockout affects proliferation and cell renewal of colon epithelia

**Figure 2:**
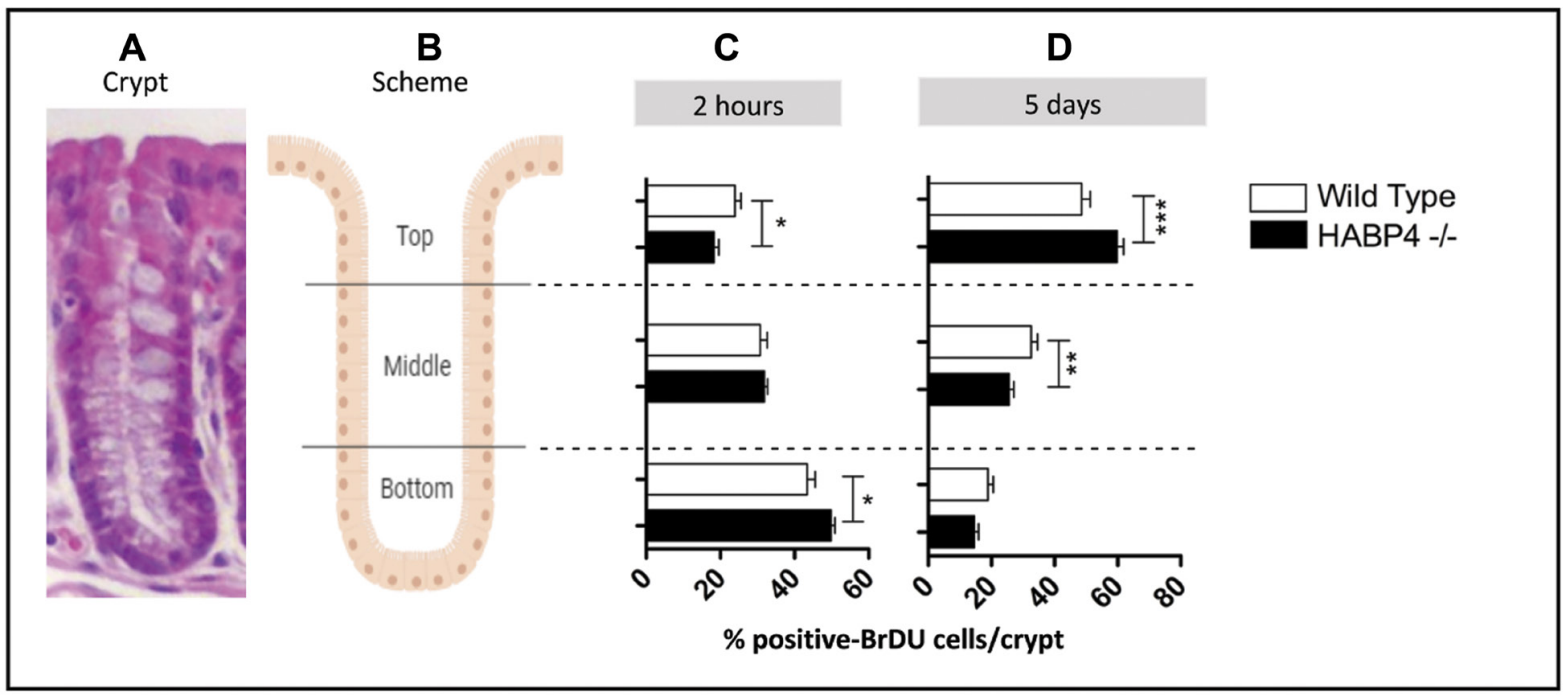
Increased proliferation in the colon epithelia of knockout mice. (**A**) histological image of the overall crypt organization, (**B**) schematic representation of the three main regions analyzed. The BrdU incorporation assay was performed with the colons of the *Habp4 –/–* animals (black) and wild-type (white), after 2 hours (**C**) and 5 days (**D**) of the injection. The cells labeled with anti-BrdU were quantified in the three compartments of the crypt, by manual counting, and at least 20 crypts per animal were counted (number of mice: control 2 h *n* = 2; HABP4 –/– 2 h *n* = 5; control 5 days *n* = 3; HABP4 –/– 5 days *n* = 5). Data are presented as mean and SD. ^*^ *p* < 0.05, ^**^ *p* < 0.005, ^***^ *p* < 0.001.

Previous data have shown that HABP4 overexpression in HEK293 cells decreases the expression of genes related to the proliferative activity [11]. To investigate *in vivo* the involvement of HABP4 in cell proliferation activity, the incorporation of bromodeoxyuridine (BrdU) by the colon epithelium cells of the wild-type and *Habp4 –/–* mice was evaluated. BrdU was injected intraperitoneally into control and *Habp4 –/–* mice, and the animals were sacrificed 2 hours or 5 days after injection. The number and position of BrdU-positive cells in the colon's crypts were evaluated with an anti-BrdU antibody and the crypts were divided into three equal sections: top, middle and bottom, as described in the materials and methods (Figure 2A and 2B).

After two hours of BrdU injection, *Habp4 –/–* mice’s (*n* = 5) colons exhibited a greater number of BrdU positive nuclei per crypt compared to wild-type animals (*n* = 2) (*p* = 0.0145; Figure 2C), indicating that the absence of HABP4 protein disrupts cell division in the colon epithelium. To follow up the path of the fastest dividing cells, the colon tissue after 5 days of BrdU injection was analyzed (Figure 2D). At that time, in both knockout (*n* = 5) and wild-type animals (*n* = 3), the number of proliferating cells at the crypt bottom decreased, showing that cells migrated towards the top of the crypt in response to the continuous cell division that occurs at the base. However, the number of BrdU positive cells was lower in the middle and bottom of the crypts of *Habp4 –/–* animals compared to wild-type. Most of the cells labeled with BrdU in *Habp4 –/–* mice were detected already at the top of the crypt, with *p*-values (P) of 0.0049 and 0.0002 to middle and top, respectively (Figure 2D).

The colon crypt cells of the *Habp4 –/–* mice exhibited a higher rate of proliferation at the bottom coupled to a faster turnover of the epithelial colon cells. Most likely, the high dynamic of the colon tissue balances the increased proliferation of epithelial cells by a more efficient shedding and, thus, maintaining a normal colon architecture in the knockout mice.

**Figure 3:**
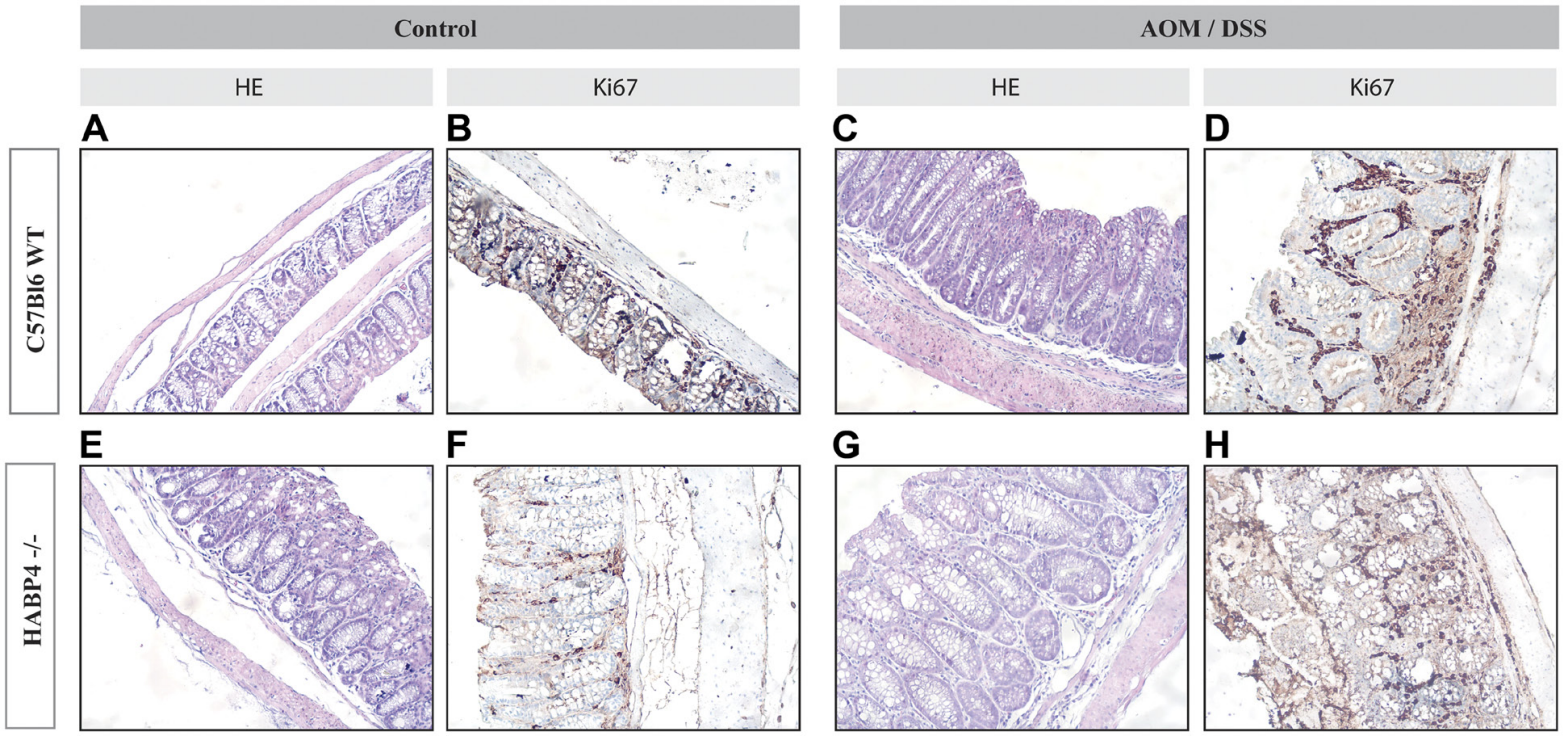
Histological sections of colon tissues of wild-type and *Habp4* –/– mice. Histological sections of wild-type mice (**A**–**D**) or *Habp4 –/–* mice (**E**–**H**), were stained with hematoxylin and eosin (A, C, E, G) or immunostained using anti-Ki-67 antibody (B, D, F, H) to assess the proliferative activity of tissues. The immunostaining of the cuts was revealed by DAB.

These findings and interpretations were corroborated by the colon histochemical analysis, which revealed no obvious changes in differentiated colon epithelial cells, such as goblet cells (Figure 3). These experiments also serve as a control that the knockout mice colon crypt organization is not grossly altered. Therefore, the molecular expression data presented in the next section are not a consequence of any evident histological disorganization in the knockout mice.

### Colon tumor induction and evaluation of proliferation marker expression

**Figure 4:**
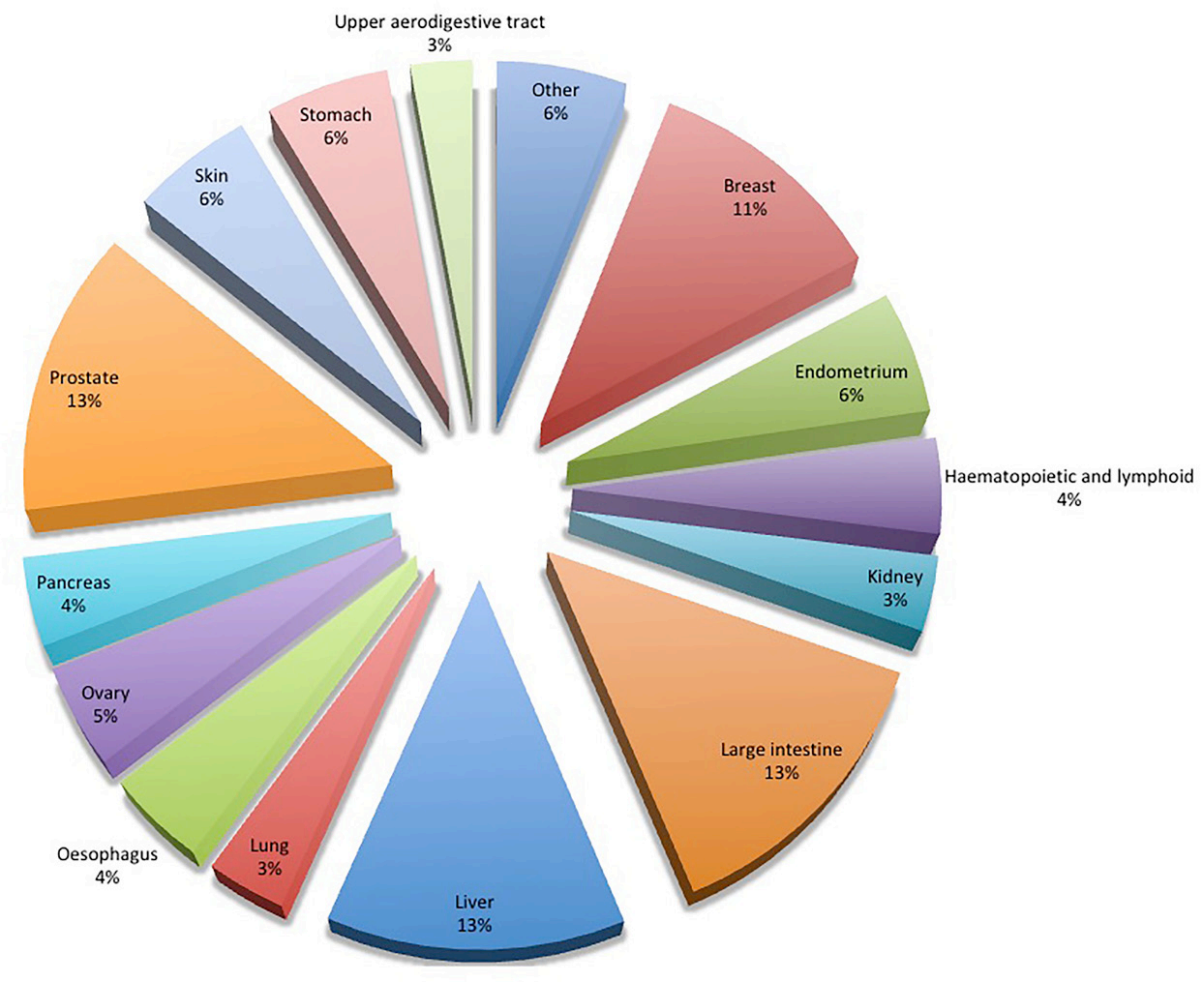
Mutations in HABP4 in different types of cancer. The COSMIC (Catalog of Somatic Mutations in Cancer) database shows the percentage of mutations in the HABP4 gene in several types of cancer, with special emphasis on cancer of the intestine, endometrium and skin.

Since the gene of HABP4 is located in a region of the chromosome 9 that has linkage related to familiar colon cancer and leukemia [5, 9], we decided to explore this possible connection further. In the COSMIC database (Catalog of Somatic Mutations in Cancer) several mutations in the HABP4 gene were found, being the intestine (13%) and related digestive tract tissue cancers (additional 30%; stomach, upper aerodigestive, pancreas, oesophagus, liver) the tissues with the highest incidence of mutations (43% total) (Figure 4). Altogether, these data suggest that HABP4 may be involved in colon cancer. To investigate this relationship, control wild-type and *HABP4 –/–* mice were subjected to colon tumors induction by colitis.

**Figure 5:**
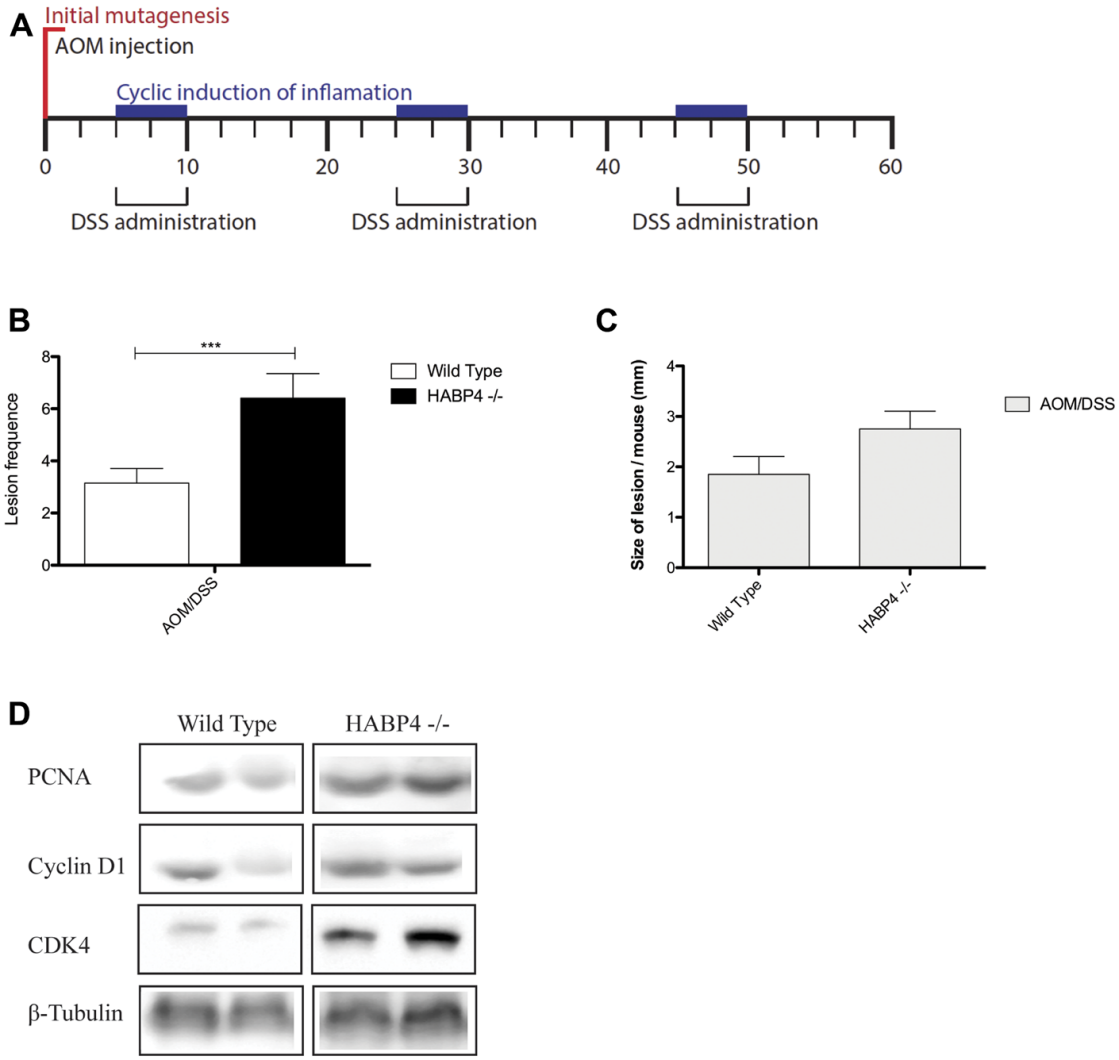
Colon tumor induction in control and knockout mice. (**A**) Representative scheme of tumor colon induced by AOM and DSS treatment (WT control *n* = 12; WT AOM/DSS *n* = 26; HABP4 –/– control *n* = 11; HABP4 –/– AOM/DSS *n* = 22); Quantification of the (**B**) number and (**C**) size of tumors between *Habp4 –/–* and wild-type mice after tumor induction treatment; (**D**) Western blot analysis of proteins involved in cell proliferation. Error bars are SD. ^***^ *p* < 0.0001.

Wild-type and *Habp4 –/–* mice were treated with the carcinogen azoxymethane (AOM), in a single dose intraperitoneally. Three days later they were exposed to three cycles of 2,5% dextran sodium sulfate (DSS) in drinking water, an irritating agent of the colon and rectum mucosa, for 5 days followed by 15 days of free treatment, as depicted in Figure 5A. The combination of both reagents decreases the colon and rectal carcinomas latency, mimicking the formation of tumors in animals [30].

At the end of treatment, the animals were sacrificed and their colon analyzed in regarding the number (Figure 5B) and maximum tumor size (Figure 5C) of tumors. Neither wild-type nor *HABP4 –/–* mice submitted to control treatment (PBS injection followed by tap water changes in the same day of DSS treatment) developed tumors. After AOM/DSS treatment, *HABP4 –/–* mice presented a higher number of tumors (Figure 5B, *p* = 0.0034), then wild-type mice. These tumors were also larger when compared to control animals that have normal HABP4 expression (Figure 5C, *p* = 0.1437).

The portion of colon without tumor tissue of treated animals was used to quantify the expression level of proteins involved in proliferative processes, to corroborate with incorporation BrdU assay. It was observed that Cyclin D1, PCNA, and CDK4 were overexpressed in the absence of HABP4 in comparison to control animals, confirming that HABP4 influences proliferative events (Figure 5D).

### HABP4 absence increases proliferation also in the HCT116 cell KO model

**Figure 6:**
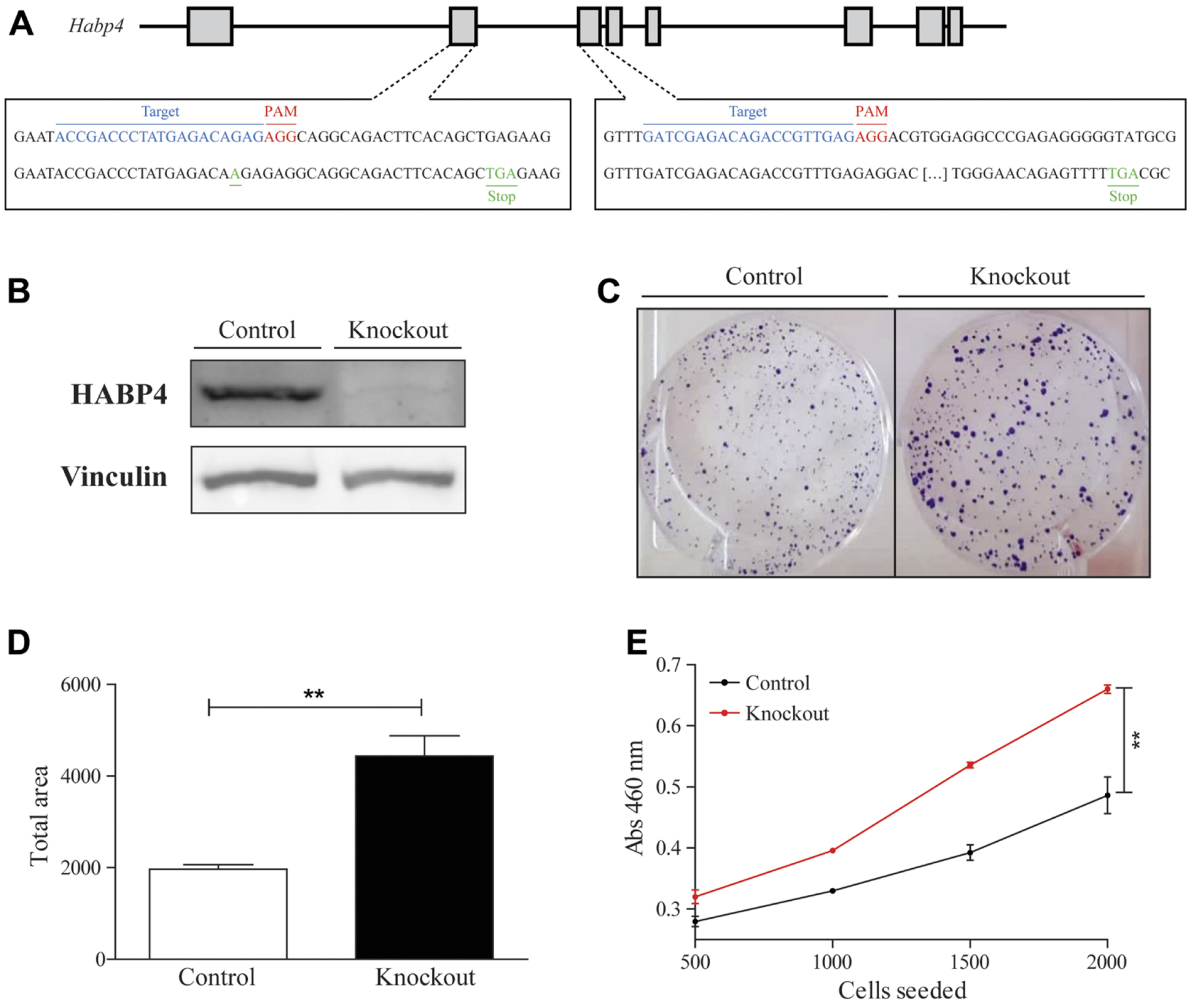
HABP4 knockout in HCT 116 cell model. (**A**) Representative knockout scheme; (**B**) Western blot analysis of HABP4 in control and knockout cells; (**C**) Clonogenic assay comparing control and knockout cells after 7 days of growth; (**D**) Statistic analysis of the clonogenic assay, *p* < 0.005; (**E**) Proliferation cell counting Kit -8 assay. Error bars are SEM. ^**^ *P* < 0.005.

Next, we asked if the role of HABP4 in the mouse model would be conserved in the human cell model. To answer this, the *HABP4* gene was also knocked out in HCT116 cells by CRISPR/Cas9 technique. The selected clone presented the insertion of an adenine in the exon 2 and a thymine in the exon 3, which causes frame-shifts leading to premature stop codons (Figure 6A). In addition to confirmation by DNA sequencing, western blot analysis showed that HABP4 protein was no longer detectable, confirming the knockout at the protein level (Figure 6B).

To evaluate if the absence of HABP4 interferes with the cell proliferation as well as does in *HABP4 –/–* mice, it was performed the clonogenic assay between HCT116 wild-type and HCT116 *Habp4 –/–*. It was observed that the HABP4 knockout had increased colony formation, presenting more and larger colonies (Figure 6C), showing that the HCT116 *Habp4 –/–* had a proliferative rate of 4,440 ± 446, which is approximately twice as high as the control cells (1,970 ± 99.9; *p* = 0.0057; Figure 6D).

Cell proliferation was also evaluated with the Cell Counting Kit 8 (CCK-8), which monitors the formazan generation. In this assay, it was observed that the HCT116 *HABP4 –/–* cells metabolized more tetrazolium salt, forming more formazan, than the control cells, which is correlated with a higher proliferation rate (*p* = 0.0026; Figure 6E).

### HABP4 expression in human colon and liver tissues

Colon cancer is the third most incident cancer and the second with the highest mortality [31]. The high incidence associated with the high mortality rate indicates that new strategies and targets need to be investigated for the treatment of colon cancer. As observed, HABP4 plays a homeostatic role in mice colon tissue, leading to an increasing number of tumors in its absence. It has also been shown that in the human cell model HABP4 knockout also increases cell proliferation. In this context, we wondered how would be the expression profile of the HABP4 in patients’ tissue.

**Figure 7:**
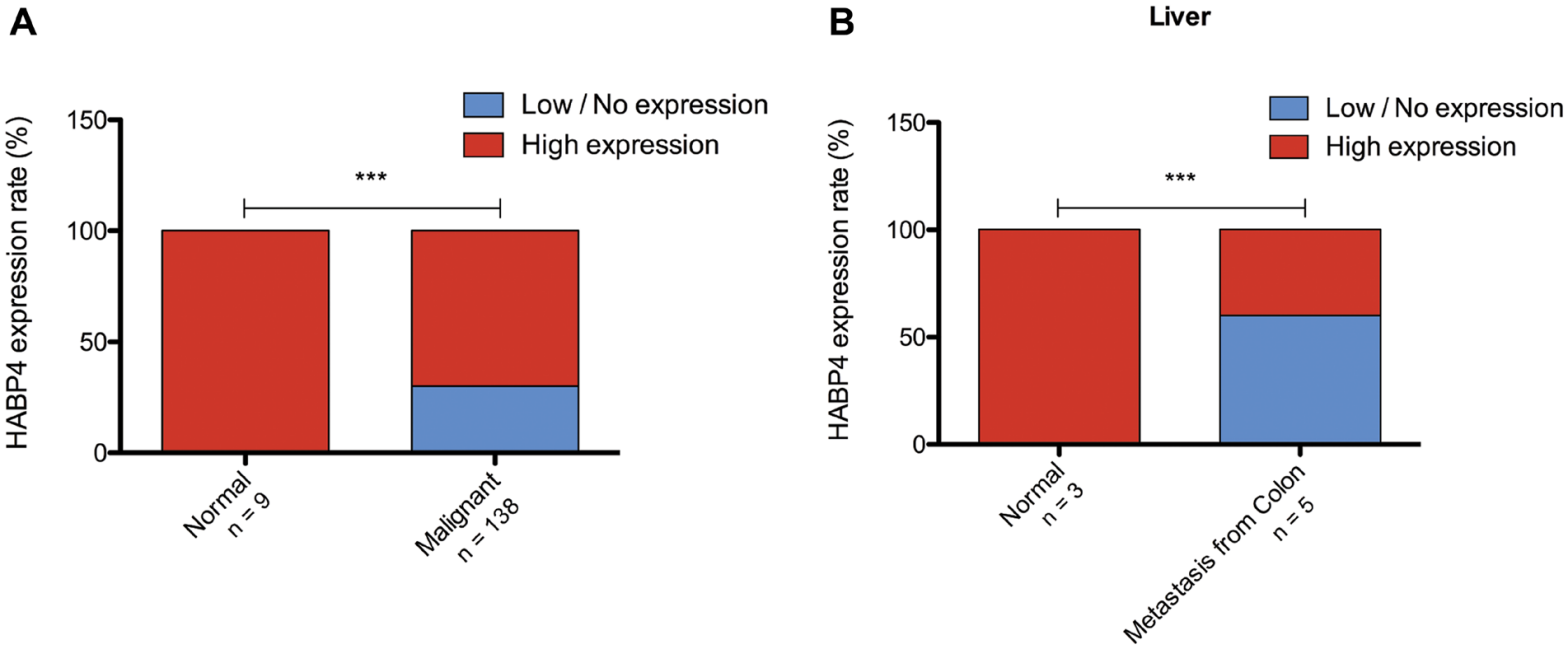
Analysis of HABP4 expression in patients’ tissue. (**A**) Immunohistochemical analysis of the HABP4 expression in normal (*n* = 9) and tumor tissues (*n* = 138) of the colon of patients; (**B**) Analysis of normal liver tissue (*n* = 3) and tumor tissue originated by colon cancer metastasis. ^***^ *p* < 0.0001.

To assess the HABP4 expression profile in colon and liver tissues, immunohistochemical assays were performed with commercially available slides containing 20 normal colon tissues, 156 malignant tissues (125 adenocarcinoma, 28 mucinous adenocarcinoma and 3 papillary adenocarcinoma), and 3 normal liver tissues and 5 tumor liver tissues in response to colon metastasis. It was observed that normal colon tissue presents a high HABP4 level. On the other hand, 30% of the tumor tissues present low or no HABP4 expression (*p* < 0.0001; Figure 7A).

The liver is one of the first foci of colon metastasis and, similarly to what was observed in colon, normal liver tissues showed a high expression of HABP4. On the other hand, its expression was decreased in tumors, with values even more expressive than the colon: 50% of the tissues had low or no expression of HABP4 (*p* < 0.001; Figure 7B).

Comparative analyses were also carried out between characteristics of physiological and histopathological with the HABP4 expression profile. As shown in Table 1, the expression of HABP4 cannot be related to gender (*p* = 0.5428), age (*p* = 0.2146), degree (*p* = 0.758) or stage (*p* = 0.095). However, the expression of HABP4 allowed discriminating between histopathological types (*p* < 0.0001).

**Table 1:** Relationship between clinical and pathological characteristics of benign (n = 20) and malignant (n = 156) colon neoplasms expressing HABP4

Cases	*n*	HABP4 expression		X^2^	Fisher´s exact test (*p*-value)
		low	high		
Gender					
Male	97	28	69		
Female	41	14	27	0.5793 (*p* = 0.446)	0.5428
Age					
≤ 60	79	27	52		
> 60	59	15	44	1.947 (*p* = 0.1629)	0.2146
Histological type					
Adenocarcinoma	120	32	88		
Mucinous adenicarcinoma	8	6	2		
Papillary adenocarcinoma	3	0	3	128.6 (*p* ≤ 0.0001)	< 0.0001
Grade					
1	33	8	25		
2 and 3	91	24	67	0.2369	0.758
TNM stage					
I + II	88	23	65		
III + IV	50	19	31	3.309 (*p* = 0.689)	0.095

## DISCUSSION

The experiments reported in this article suggest that HABP4 may represent a novel tumor suppressor in colon cancer. *HAPB4 –/–* mice have a higher chance to develop more and larger tumors when challenged with AOM/DSS then wild-type animals and the colon tissue shows an increased expression of proliferation markers, even without any treatment with AOM/DSS.

The Ki-1/57 antigen had been discovered initially as a cross-reacting, intracellular 57 kDa phosphoprotein, of the antibody Ki-1 [7], which detects the 120 kDa surface glycoprotein CD30 (=TNFRSF8), a member of the TNFR-superfamily [5, 10]. After molecular cloning by different groups and discovery associated with different molecular contexts the protein was renamed to HABP4 (=Intracellular hyaluronic acid-binding protein) [4, 9]. Although the group that discovered the biochemical, *in vitro* interaction of HABP4 with hyaluronic acid, RNA and other negatively charged molecules, could neither demonstrate the occurrence nor possible functional meaning of such interaction inside the cell or even nucleus, considering that hyaluronic acid is of extracellular localization.

At the time of its discovery not much was known on the possible functions of this protein [9]. However, several molecular features suggested that it might be a regulatory protein with some association with cancer [7, 8]. As many other oncoproteins HABP4 showed a shuttling protein localization between the cytoplasm and the nucleus, a localization to protein bodies in the cytoplasm, especially after cellular stress, co-localization with nuclear splicing speckles and other nuclear sub-structures [13], extensive posttranslational modifications, including serine/threonine phosphorylation by PKC [20], arginine methylation [18] and SUMOylations [32]. Furthermore, the amino acid sequence of HABP4 is rich in charged amino acids and the protein seems to be part of the family of so called IUPs (intrinsically unstructured proteins) [27], proteins which are known to have many interactors, frequently with regulatory functions, that tend to occupy hub positions in complex protein networks and are frequently described for their involvement in cancer related cellular processes [33].

The identification of the protein interactors of HABP4 and SERBP1, was able to shed some new light on their possible molecular functions or their involvement in cellular processes [12–14, 18, 19, 21]. HABP4 interacts with several proteins involved in transcriptional regulation, including CHD-3, DAXX, HMG, p53, and TOPORS, TIP60, among others. Furthermore, it interacts with several proteins involved in the metabolism of RNA: CIRBP, YB1, NSEP-1, SFRS9, SF2, FXRP [10].

The first concrete link that suggested some relevance of HABP4 in cancer was soon after it´s cloning [9], when FISH experiments showed that the gene of HABP4 is located in region 9q22.3-3.1 of the chromosome 9 that had been reported as a hot spot for secondary aberrations in acute myeloid leukemia. A later study in a familial form of colon cancer also was able to zoom in to a region around 9q22.2-31.2, in which aside the gene of HABP4 only three other genes are found [4], which seem to be less likely candidates for oncoproteins or tumor suppressors (ZNF367, GABBR2 and GALNT12). Although GalNAc transferase 12 gene plays a role in the initial step of the synthesis of mucine-like oligosaccharides, in the colon and other organs, somatic mutations in GALNT12 in colon cancer have been reported to be rather rare. The 9q22.3-3.1 region is in clear linkage disequilibrium with SNP haplotypes in the families with higher colon cancer risk and therefore puts HABP4 as the main candidate gene in the spotlight.

Based on these findings we generated the knockout mice (Figure 1) for the gene of HABP4 and employed an inducible colon cancer model (AOM/DSS) in the normal and knockout mice littermates to test if there are differences in the cell proliferation and other cancer related features. We found indeed that the HABP4 knockout mice in comparison with the wild type littermates tend to develop more tumors that tended to be larger (Figure 5). The higher proliferation of the bottom crypt cells 2 hours after BrdU labeling and after 5 days at the top of the crypt cells in the knockout mice confirmed the proliferative gain after HABP4 loss of function. In accordance with these findings, the tumor cells from the knockout mice had a higher expression of the proliferation markers PCNA, Cyclin D1 and CDK4.

In a microarray study with an over-expression of HABP4 in comparison to control cells with very low expression level [11], we observed a general tendency of a down-regulation of the expression of target genes in about 90% of the identified genes. Among the down regulated genes are several important regulators of cellular proliferation, including: MAP2K5, MAPK12, PLAU, PDXK, NRG1, TXLNA (alpha-taxilin). Furthermore, the cultured cells that over-express HABP4 indeed decreased proliferation. These findings together are in agreement with the findings reported here that the complete deletion of the gene HAPB4 both in the whole animal setting as well as in single cell culture increase the rate of proliferation, as would be expected from the previous over-expression studies.

Finally, we need to consider the fact that the HABP4 protein forms a family of proteins that consists in vertebrates of two members [11], with the gene CGI-55 or SERBP1 or PAI-RNABP1, being the other member. SERBP1 shares most of the characteristics of HABP4 at least partially [10]. It possesses the so called central “hyaluronic acid binding domain”, rich in charged amino acids, predominately positively charged ones, and also displays many features of an unstructured protein as HABP4 [11, 15, 17]. Most of the amino acids that are targets of the diverse post translational modifications are conserved and present also in SERBP1, which also transits between the nucleus and cytoplasm, as HABP4 [10]. Both proteins localize to Cajal bodies, nuclear splicing speckles and PML bodies [13, 14] and share several of their interacting proteins, including: CHD3, TOPORS, UBC9. RACK1, DAXX, PRMT1, TDG, and PIAS [10, 15].

A study of the general transcriptome after SERBP1 over-expression revealed, as in the case of HABP4, a predominant repression of 90% of the transcripts [11]. Again several mRNAs coding proteins involved in proliferation regulation (MAP2K5, PAK7, ODZ1, FoxP1, NDEL1, LST1) are down regulated and indeed over expression of SERBP1 diminishes the proliferation rate of the transfected cells, too, as does over-expression of HABP4.

Although many functional features tend to overlap between HABP4 and SERBP1, in relation to cancer the data so far point in opposite directions, since SERBP1 has been reported to be over-expressed in several cancer settings, including ovary, breast, colon, prostate, glioblastoma and lung cancer [34]. The higher expression levels of SERBP1 has been associated to higher grading of the tumor stages, making SERBP1 a prognostic predictor candidate [24, 34, 35] and candidate gene involved in the tumorigenesis process and resistance to anti-cancer drugs [34, 36].

Here, on the other hand, we show for HABP4 the opposite: its lack of function (gene knockout in animals and cells), or more specifically, its diminished protein expression, is indicative of the tumor status of the tissue, since in Tissue Microarrays, 30% of the cancer tissues showed low or no expression of HABP4. Also in 50% of the metastasis of liver cancer had diminished or absent expression of the HABP4 protein. 43% of the mutations found for HABP4 in cancer, are found in samples from patients with cancers in the digestive tract: intestine (13%), stomach (6%), esophagus (4%), upper aerodigestive tract (3%), pancreas (4%) and liver (13%) (Figure 4). the TMA by chi-square or Fisher’s exact test, as indicated in Table 1.

In summary, our new data from the HABP4 mouse and human cell culture knockout models, together with expression data from colon cancer patient samples and literature data, suggest that HABP4 is a new tumor suppressor candidate. The main phenotype that contributes to tumorigenesis upon HABP4´s lack of function is an increase in cell proliferation and increase of several proliferation marker proteins. Although the detailed underlying molecular mechanisms need to be further characterized in future experiments, HABP4 seems to be an interesting candidate to explore new diagnostic, prognostic and possibly even therapeutic avenues in colon cancer.

## MATERIALS AND METHODS

### HABP4 knockout in a mouse model

HABP4 gene knockout mice were generated through the CRISPR/Cas9 genome editing tool by Model Organism Laboratory at LNBio/CNPEM. A 20 nucleotides (nt) guide sequence, specific for the first *Habp4* exon, was designed as proposed by Hsu and co-workers [37] and cloned into the bicistronic expression vector px330, which contains a U6 promoter-driven single guide RNA (sgRNA) and CBh promoter-driven human codon-optimized *Streptococcus pyogenes* Cas9 (*hSp*Cas9) expression cassettes.

The circular plasmid (px330-*Habp4*-sgRNA) was microinjected into the pronuclei of C57BL/6J zygotes. Microinjected embryos were implanted into pseudopregnant CB6F1 (BALB/c x C57BL/6J) foster mothers. Mice were genotyped by PCR amplification from tail genomic DNA using Habp4-PCR forward primer (5′- CTTGCAGCCTCCCTGAGTTTCTGGT-3′) and Habp4-PCR reverse primer (5′- CAGCTCCTCCACGAAACCAGGAATG-3′), followed by T7 Endonuclease I assay (T7EI) and/or NruI restriction enzyme digestion since the 20-nt target sequence contains an endogenous site for this enzyme. To identify the type of mutation in these animals, 1200-bp PCR fragment that contains the region of the mutation was cloned into the pGEM-T Easy vector for DNA sequencing.

The knockout animals were mated with wild-type for the generation of a heterozygous littermate strain. Then, these animals were intercrossed and the offspring genotyped. This mating generated both knockout and wild-type mice, which originated the next offspring used in the experiments. All animal procedures were approved by the Institutional Animal Care and Use Committee (CEUA/UNICAMP, protocol 2661-1) and (CEUA/CNPEM, protocol 49).

### T7 Endonuclease I genotyping assay

For the T7EI assay, the 1200-bp fragment was purified by PCR purification kit (Qiagen, Germantown, MD, USA) and submitted to the assay with T7 Endonuclease I enzyme (New England Biolabs, Whitby, ON, Canada), which recognizes and cleaves non-perfectly matched DNA. As a positive control, a 1:1 mixture of wild-type and mutant DNA fragment was used.

Briefly, 200 ng of founder’s PCR fragment or the mixture of mutant and wild-type DNA were denatured at 95°C for 10 minutes, followed by annealing during cooling at –2°C per second until 85°C, and slow cooling at –0.1°C per second until 25°C. After the formation of DNA heteroduplexes, they were incubated with 5 U of T7EI enzyme at 37°C for 30 minutes. Then, the reaction was resolved by 1% agarose gel electrophoresis. The undigested fragment must contain 1200 bp and after cleavage, bands of approximately 500 bp and 700 bp are released.

### Immunohistochemistry and tissue micro arrays

The mice’s paraffin-embedded colons were cut into 5 μm sections and extended on silanized glass slides for immunohistochemical processing. The cuts were deparaffinized in 3× 100% xylol batteries and rehydrated in a decreasing series of ethanol (100% ethanol 3 times, 70% and 60%) followed by water for 5 minutes. Endogenous peroxidase was blocked by incubation with 3% hydrogen peroxide, followed by recovery of epitopes in 10 mM sodium citrate solution, pH 6, and then allowed to cool for another 30 minutes at room temperature. The blockade was performed with 5% skimmed milk in TBST (20 mM Tris-HCl pH 7.6, 150 mM NaCl, 0.1% Tween-20) for 30 minutes at room temperature.

In Figure 1D we used as primary antibody (HPA055969 – Sigma Aldrich (St. Louis, MO, USA), 1:100 dilution) incubation at 37°C for 1 hour, and then washed with TBST 3 times. In Figure 4 we used as primary antibody Ki-67 (pure culture supernatant) or detection of proliferating cells.

The Advance development system (K4066 – DAKO-Agilent, Sta Clara, CA, USA) was used followed by the chromogen DAB (3,3′-diaminobenzidine tetrahydrochloride), and Mayer's hematoxylin was used as a counter mark. Subsequently, the slides were rinsed in 1% ammoniacal water and then they were dehydrated with 100% alcohol for 3 times and 3× 100% xylol batteries. Specificity of the HABP4 antibody was verified by at least two of the authors (TDMH and CC), both blinded to tissue features. HABP4 and Ki-67 staining were analyzed through the intensity of staining. The Leica DM5500B microscope was used to visualize staining for immunohistochemistry.

The Tissue Micro Arrays were commercially purchased from US Biomax, Inc.(Rockville, MD, USA) (CO702B and BCP051110c), and it was evaluated 20 normal colon tissues, 125 adenocarcinoma, 28 mucinous adenocarcinomas, and 3 papillary adenocarcinomas. Also, 3 normal liver tissues and 5 tumor liver tissues in response to colon metastasis were evaluated.

The assessment was visual for each tissue, estimating the percentage of positive tumor cells and the intensity of the staining. The percentage was classified as 0 = no positive cells; 1 = 1 to 30% positive cells; 2 = 31 to 50% positive cells; 3 = 51 to 80% positive cells; 4 = 81 to 100% positive cells. The intensity was graded as been 0 = negative; 1 = weak coloring; 2 = moderate color; 3 = strong coloring. The final score was calculated by adding both the percentage of positive cells and the intensity of immunostaining, which ranged from 0 to 7. For statistical purposes, the cases scored less than 6 were considered as low expression and greater than 6, high express.

### BrdU incorporation

Twelve-week-old mice were intraperitoneally injected with 60 mg/Kg bromodeoxyuridine (BrdU) and then sacrificed after 2 hours or 5 days of injection. Afterward, the colon of these animals was removed and parafinized using the Swiss-Roll method [38]. The immunohistochemistry was performed with anti-BrdU antibody from Biolegend (San Diego, CA, USA), as described above and BrdU-positive cells were quantified from at least 20 crypts per animal, where each crypt was divided into 3 equally sized sections: top, middle, and bottom. To assess the percentage of BrdU-positive cells in each session, the number of positive cells in each crypt was considered as 100%. Number of mice: 2 h control *n* = 2; 2 h HABP4 -/- *n* = 5; 5 days control *n* = 3; 5 days HABP4 -/- *n* = 5.

### AOM/DSS colitis carcinogenesis induction

Eight-week-old mice weighing approximately 25g were intraperitoneally injected with 10 mg/Kg azoxymethane (AOM, Sigma-Aldrich^®^). After three days, three cycles of 2.5% (w/v) dextran sodium sulfate (DSS, MW 36–50 KDa, MP Biomedical, Inc) administered in the drinking water for 5 days followed by 14 days without treatment. 10 days after the last cycle ended, the mice were sacrificed [30, 39]. For the AOM/DSS protocol 26 wild type (WT) and 22 HABP4 –/– mice were submitted to the AOM/DSS treatment and 12 WT and 11 HABP4 –/– mice were used as controls. Two independent experiments were performed. Before tissue extraction, the number and the size of intestinal tumor lesions were evaluated. Animals were apart for each experiment. Tumor lesions were extracted for Western blotting and colon tissue removed for immunohistochemistry.

### Western blotting

To perform Western blot experiments, 50 μg of tissue lysate samples were separated using SDS-PAGE and transferred to a 0.45 μm nitrocellulose membrane using the wet system from Bio-Rad (Hercules, California, USA). The membrane was blocked for 1 hour at room temperature using 5% skimmed milk in TBST (see above). For immuno-detection of proteins, membranes were incubated overnight at 4°C with primary antibodies against HABP4 (rabbit, polyclonal, 1:1000 dilution), PCNA (mouse, monoclonal, 1:1000 dilution), Cyclin D1 (rabbit, monoclonal, 1:1000 dilution), or CDK4 (mouse, monoclonal, 1:1000 dilution), diluted in TBST. Three washes of 5 minutes with TBST solution were done and membranes were incubated for 1 hour at room temperature with secondary antibodies anti-rabbit IgG or anti-mouse IgG (Milford, Massachusetts, EUA) (1:5000 dilution), in TBST containing 5% skimmed milk. Three washes of 5 minutes with TBST were done and membranes were developed using the enhanced ECL method using Chemi Doc Imaging System from Bio-Rad. All directly compared samples of e. g. Cyclin D1 from wild-type and HABP4-/- mouse samples (see Figure 5D, second line) were run out side by side on the same gel and blotted to the same blot to guarantee equal processing, exposure times, etc. Only this way it can be guaranteed that the samples can be compared in a semi-quantitative manner.

### Cell culture

The human colon cell line HCT 116 and HCT 116 *Habp4 –/–* cells were cultured in DMEM medium supplemented with 10% of fetal bovine serum at 37°C, 5% CO_2_ and 95% humidity. These cells were used to generate the knockout cells of HABP4.

### HABP4 knockout in HCT 116 cellular model

To knockout HABP4 from HCT 116 cell line, the CRISPR/Cas9 system from *Santa Cruz Biotechnology (*sc-408202 and sc-408202-HDR; Dallas, TX, USA) was used. The clone selection was due through puromycin resistance followed by cell sorting with the puromycin-survived cells. To validate the knockout, DNA was sequenced with primers surrounding the sgRNA region, and through western blot analyisis using anti-HABP4 antibody (ab178593, Abcam, Cambridge, MA, USA).

### Clonogenic assay

1500 cells of each cell line, HCT 116 control and HCT 116 Habp4 –/–, were seeded into 6-well dishes and cultured for 7 days. After incubation time, the medium was gently removed and the cells were fixed and stained with a solution of crystal violet (0.05% w/v), 37% formaldehyde (1%), and methanol (1%) for 30 minutes at room temperature. The difference was measured by the total area of the colonies, considering *p* < 0.05, using ImageJ. The experiment was performed at least three times.

### Cell counting kit – 8 (CCK-8) assay

Cell proliferation was also monitored by colorimetric water-soluble tetrazolium salt (WST-8) assay using a Cell Counting “Kit-8” (Dojindo, Rockville, MD, USA) according to the manufacturer’s instructions. The metabolic activity of the cells reduces the WST-8 in formazan, conferring an orange color to the culture medium. This coloration was measured by absorbance at 460 nm, which is proportional to the number of cells.

500, 1000, 1500, and 2000 cells of each cell line (HCT 116 control and HCT 116 Habp4 –/–) were seeded into 96-well dishes and cultured for 3 days. After, 10% of the culture medium volume of WST-8 reagent was added, and the cells were incubated with the reagent for 2 hours at 37°C, protected from light, for later absorbance analysis. The CCK-8 assay was done three times with independent cell culture samples.

### Statistical analyses

Statistical analyses were performed using GraphPad Prism software. Two-tailed unpaired *t*-test was applied to BrdU incorporation, clonogenic assay, and size and frequency of lesions in the mice colon, considering *p* < 0.05. The CCK-8 assay was analyzed by ANOVA and the TMA by chi-square or Fisher’s exact test, as indicated in Table 1.
